# T-2 Toxin Induces Oxidative Stress at Low Doses via Atf3ΔZip2a/2b-Mediated Ubiquitination and Degradation of Nrf2

**DOI:** 10.3390/ijms22157936

**Published:** 2021-07-25

**Authors:** Xiaoxuan Chen, Peiqiang Mu, Lang Zhu, Xiaoxiao Mao, Shuang Chen, Huali Zhong, Yiqun Deng

**Affiliations:** 1 Guangdong Provincial Key Laboratory of Protein Function and Regulation in Agricultural Organisms, College of Life Sciences, South China Agricultural University, Tianhe District, Guangzhou 510642, China; xuanzi@scau.edu.cn (X.C.); mpeiqiang@scau.edu.cn (P.M.); 20192003022@stu.scau.edu.cn (L.Z.); mao706@stu.scau.edu.cn (X.M.); shuangchen@stu.scau.edu.cn (S.C.); 20202003035@stu.scau.edu.cn (H.Z.); 2 Key Laboratory of Zoonosis of Ministry of Agriculture and Rural Affairs, South China Agricultural University, Guangzhou 510642, China; 3 Guangdong Laboratory for Lingnan Modern Agriculture, South China Agricultural University, Guangzhou 510642, China

**Keywords:** T-2 toxin, ROS, ATF3ΔZip2a/2b, Nrf2, ubiquitination, degradation

## Abstract

T-2 toxin is mainly produced by *Fusarium* species, which is an extremely toxic mycotoxin to humans and animals. It is well known that T-2 toxin induces oxidative stress, but the molecular mechanism is still unknown. In this study, we found that T-2 toxin significantly promoted reactive oxygen species (ROS) accumulation in MCF-7 cells at low doses which maintains cell viability at least 80%. Further analysis showed that T-2 toxin downregulated the expression of the master regulator of antioxidant defense gene, nuclear factor erythroid 2-related factor (*Nrf2*), and its targeted antioxidant genes. Overexpression of Nrf2 or its target gene heme oxygenase 1 (HO1) significantly blocked the ROS accumulation in MCF-7 cells under T-2 toxin treatment. Moreover, we found that T-2 toxin downregulated the antioxidant genes via inducing the expression of ATF3ΔZip2a/2b. Importantly, overexpression of ATF3ΔZip2a/2b promoted the ubiquitination and degradation of Nrf2. Altogether, our results demonstrated that T-2 toxin-induced ROS accumulation via ATF3ΔZip2a/2b mediated ubiquitination and degradation of Nrf2, which provided a new insight into the mechanism of T-2 toxin-induced oxidative stress.

## 1. Introduction

T-2 toxin is a type A trichothecene mycotoxin produced by *Fusarium* species, which is stable and widely distributed and has threatened livestock production and human health for decades [1,2]. Previous studies showed that T-2 toxin had a lot of toxic effects, such as inhibiting DNA and protein synthesis [3], inducing DNA damage and cell apoptosis [4], interfering with the metabolism of membrane phospholipids and disrupting energy metabolism and gut microbiome [5,6]. It was shown that oxidative stress was associated with several toxic effects of T-2 toxin [7]. However, the molecular mechanism of T-2 toxin-induced oxidative stress is still unknown.

Oxidative stress arises from an imbalance between generation and elimination of ROS, leading to the activation or inhibition of genes encoding defensive enzymes, transcription factors and structural proteins [8,9]. ROS, including singlet oxygen (O_2_), superoxide radical (O^–^), hydrogen peroxide (H_2_O_2_) and hydroxyl radical (HO), have been considered to be the second messenger independent of oxidative stress and serves as a signal of cell proliferation, necrosis and apoptosis [10]. Accumulation of ROS may induce cell oxidative injury, such as DNA damage, oxidation of proteins and lipid peroxidation [11]. Previous studies showed that Poly (ADP-ribose) polymerase-1 (PARP-1), NADPH1 oxidase p22phox (CYBA), Myeloperoxidase (MPO) are involved in ROS production [12,13,14]. E.g., overexpression of PARP-1 caused ROS-induced damage which is dependent on the over-activation of glycolysis upstream pathways, while downregulation of PARP-1 limited-oxidative-stress-induced injury [15,16]. Besides the ROS production system, the cellular ROS levels are also dependent on the activity of antioxidant system. Numerous studies showed that the glutathione system, heme system and superoxide scavenger system are involved in enzymatic and transcriptional activity which reduced the oxidative stress [17,18]. Due to the activity of oxidants systems and antioxidant systems which control the production of ROS, cells maintain the oxidative homeostasis. However, it is unclear which ROS-related genes are involved in T-2 toxin-induced oxidative stress.

A series of antioxidant defense genes and cellular signaling pathways have been identified under oxidative stress. Among them, the Nrf2 is considered a master regulator of antioxidant defense genes [19]. Nrf2 is a member of the bZIP transcription factor family with a high sensitivity to oxidative stress, which regulates the expression of an array of detoxifying and antioxidant defense genes [20,21]. Under normal conditions, Nrf2 interacts with the cytosolic repressor protein Kelch-like ECH-associated protein 1 (Keap1) in the cytoplasm and maintains at low levels because of the ubiquitination and degradation [22]. While under oxidative stress conditions, Nrf2 dissociates from Keap1 and translocates into the nucleus to activate the transcription of a variety of antioxidant and detoxification genes. The activated genes regulated by the Nrf2 pathway include numerous genes involved in general stress response (heme oxygenase and thioredoxin), protection from electrophiles (glutathione synthesis and superoxide dismutase) and xenobiotic disposition (glutathione–S–transferases and quinone reductases). It has been reported that Nrf2 can regulate the cellular levels of ROS to control the cellular processes, such as cell proliferation and differentiation [23]. However, the role of Nrf2 in T-2 toxin-induced oxidative stress is still unknown.

ATF3 (activating transcription factor3) is a stress response gene that is induced during the cellular responses to many stress signals including adipokines, cytokines, hypoxia, mycotoxins and chemokines [24,25]. The expression of ATF3 is transient and plays a pivotal role in regulating the expression of cell-cycle regulators and tumor suppressors, DNA repair and apoptotic genes [26]. Overwhelming evidence suggests that ATF3 plays a prominent role in controlling the cell cycle progression, inflammation, apoptosis, endoplasmic reticulum stress, oxidative stress and diseases [27,28]. Recent studies suggest that ATF3 is important in oxidative stress response. The overexpression of ATF3 leads to the accumulation of depolarized mitochondria, loss of cell viability and increased production of mitochondrial ROS [26]. It has been reported that ATF3 represses PTEN-induced kinase 1 (PINK1) mRNA synthesis to dysregulate mitochondrial homeostasis [29]. However, few reports are available about the role of ATF3 in the process of T-2 toxin-induced oxidative stress.

ATF3 and Nrf2 play pivotal functions in oxidative stress response, but the roles of ATF3 and Nrf2 in T-2 toxin-induced oxidative stress remain unknown. In this study, we clarified the molecular mechanisms of T-2 toxin-induced oxidative stress in MCF-7 cells. Our results indicated that the ATF3ΔZip2a/2b mediated downregulation of Nrf2 was critical in T-2 toxin–induced ROS accumulation, which provides new insight into the toxicology of T-2 toxin.

## 2. Results

### 2.1. T-2 Toxin Promoted Ros Accumulation in Mcf-7 Cells at Low Doses

**Figure 1:**
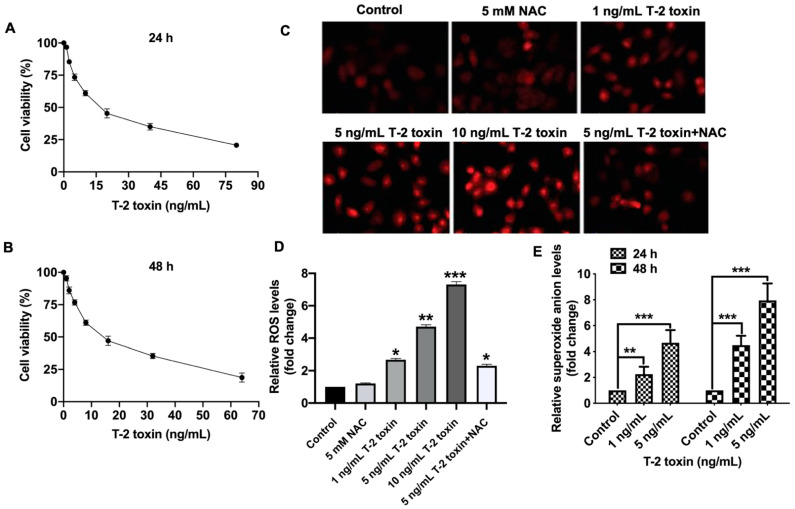
T-2 toxin promoted ROS accumulation in MCF-7 cells. (**A**,**B**) Cell viability of MCF-7 cells under T-2 toxin treatment. MCF-7 cells were treated with gradient concentrations of T-2 toxin for 24 h (**A**) and 48 h (**B**). The cell viability was measured by Cell Counting Kit-8 assay. (**C**) ROS staining in MCF-7 cells under T-2 toxin treatment. The MCF-7 cells were treated with different concentrations of T-2 toxin (1, 5 or 10 ng/mL) for 24 h. The ROS were stained DCFH-DA and observed by fluorescence microscopy. To eliminate ROS induced by T-2 toxin, 5 mM ROS scavenger (NAC) was applied 18 h before T-2 toxin was added. (**D**) The fold change of intracellular ROS levels under T-2 toxin treatment in MCF-7 cells. MCF-7 cells were treated with 1 ng/mL and 5 ng/mL T-2 toxin for 24 h, ROS were stained with DCFH-DA and measured by the absorbance with excitation at 488 nm and emission at 525 nm. (**E**) The fold change of superoxide anion levels under T-2 toxin treatment in MCF-7 cells. MCF-7 cells were treated with 5 ng/mL T-2 toxin for 24 h and 48 h, ROS were stained with DHE and measured by the absorbance with excitation at 488 nm and emission at 525 nm. Each value represents the mean ± SEM of at least three independent experiments. Significant differences were defined as * *p* < 0.05, ** *p* < 0.01 or *** *p* < 0.001.

**Figure 2:**
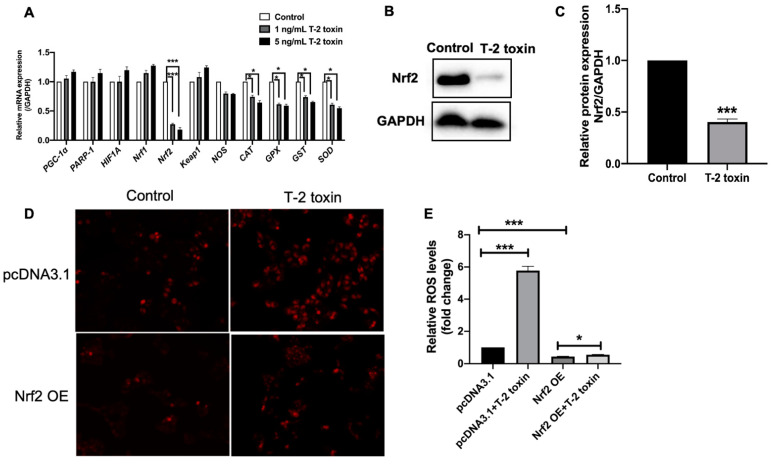
Nrf2 was significantly downregulated under T-2 toxin treatment and the ROS accumulation was attenuated in Nrf2 overexpression MCF-7 cells. (**A**) Fold changes of mRNA levels of oxidative stress-responsive genes (*PGC-1α*, *PARP-1*, *HIF1A*, *Nrf1*, *Nrf2*, *Keap1*, *NOS*, *CAT*, *GPX*, *GST* and *SOD*) in MCF-7 cells treated with 1 ng/mL or 5 ng/mL T-2 toxin for 24 h. (**B**) The Western blot of Nrf2 in MCF-7 cells treated with 5 ng/mL T-2 toxin for 24 h. (**C**) Quantitative analysis of Nrf2 protein levels in panel B. (**D**,**E**) The ROS levels were measured by DCFH-DA staining. MCF-7 cells were transfected with Nrf2 or pcDNA 3.1 plasmids and treated with or without 5 ng/mL T-2 toxin for 24 h. Significant differences were defined as * *p* < 0.05, *** *p* < 0.001.

To investigate the mechanism of oxidative stress under T-2 toxin treatment, the cytotoxicity of T-2 toxin and ROS accumulation were studied in human breast adenocarcinoma cell line MCF-7. The cytotoxicity of T-2 toxin in MCF-7 cells was measured by CCK-8 assay. After incubation with T-2 toxin for 24 h or 48 h, the cell viabilities of MCF-7 cells were significantly decreased, with the half maximal inhibitory concentration (IC_50_) values of 16.3 ng/mL and 13.7 ng/mL, respectively (Figure 1A,B). To alleviate the cell death of T-2 toxin, two low doses (1 ng/mL and 5 ng/mL), which maintained cell viability at least 80%, were used in subsequent assays. To determine the ROS levels under T-2 toxin treatment, MCF-7 cells were treated with 1, 5 or 10 ng/mL T-2 toxin for 24 h, and then the ROS were stained with 2′,7′-dichlorodihydrofluorescein diacetate (DCFH-DA) (Figure 1C,D) and dihydroethidium (DHE) (E). As shown in Figure 1C,D, T-2 toxin induced the intracellular ROS accumulation in a concentration-dependent manner and the ROS scavenger (NAC) could obviously alleviate the ROS accumulation. The superoxide anion levels were further quantified by DHE staining with up to eight-fold increase under 5 ng/mL T-2 toxin treatments for 48 h (E). Altogether, T-2 toxin significantly induced ROS accumulation in MCF-7 cells even at low doses with 80% cell viability.

### 2.2. Nrf2 Was Critical for T-2 Toxin-Induced Ros Accumulation

The intracellular ROS levels are dependent on the balance of ROS production and elimination. To explore the molecular mechanisms of ROS accumulation induced by T-2 toxin, the expression of main genes which were involved in ROS production and elimination under T-2 toxin treatment was determined by quantitative RT-PCR. The results showed that the expression of a master regulator of antioxidant defense genes, *Nrf2*, was downregulated by four and six-fold under 1 ng/mL and 5 ng/mL T-2 toxin treatments, respectively (Figure 2A). Consistently, the expressions of antioxidant genes, including oxide synthase (*NOS*), catalase (*CAT*), glutathione peroxidase (*GPX*), glutathione S-transferases (*GST*), Superoxide dismutase (*SOD*), were downregulated by approximately 1.5-fold (Figure 2A). However, the expressions of ROS production genes, including Peroxisome proliferator-activated receptor-gamma co-activator-1alpha (*PGC-1α*), *PARP-1*, hypoxia-inducible factor 1α (*HIF1A)* and nuclear factor erythroid 2 related factor-1 (*Nrf1*), had no significant changes after T-2 toxin treatment (Figure 2A). The downregulation of Nrf2 was further confirmed on the protein level by Western blot (Figure 2B,C). To demonstrate the role of Nrf2 in the progress of T-2 toxin-induced ROS accumulation, the ROS levels were analyzed in Nrf2 overexpressed MCF-7 cells. As shown in Figure 2D,E, Nrf2 overexpression significantly blocked the ROS accumulation induced by 5 ng/mL T-2 toxin. Together, these data suggested that the downregulation of Nrf2 was critical for ROS accumulation under T-2 toxin treatment.

### 2.3. T-2 Toxin Downregulated the Expression of Antioxidant Genes by Nrf2

**Figure 3:**
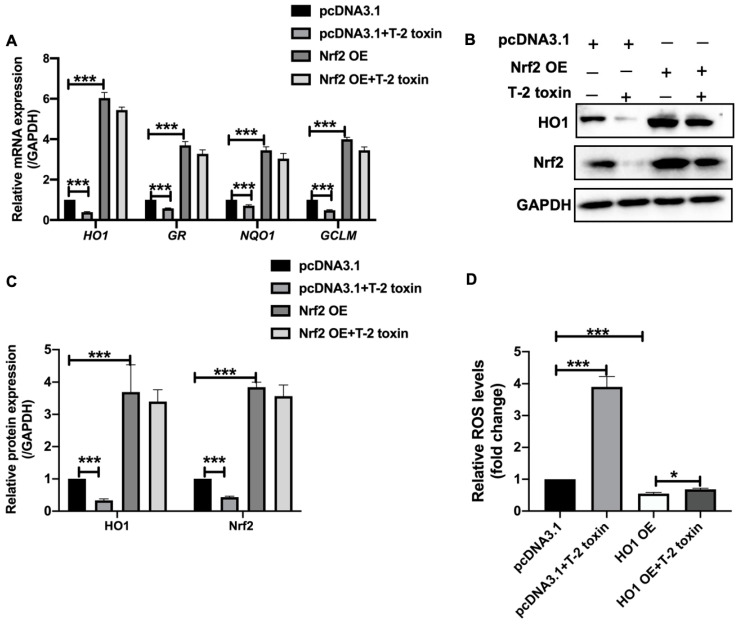
T-2 toxin downregulated the expression of antioxidant genes by Nrf2. (**A**) Fold changes of mRNA levels of the antioxidant genes (*HO1*, *GR*, *NQO1* and *GCLM*) in Nrf2 overexpressed MCF-7 cells, treated with or without 5 ng/mL T-2 toxin for 24 h. (**B**) Western blot analysis of HO1 protein levels in Nrf2 transient expressed MCF-7 cells under 5 ng/mL T-2 toxin treatment. (**C**) The Band intensities in panel B were quantified and normalized to GAPDH. (**D**) ROS levels in HO1 overexpressed MCF-7 cells treated with or without 5 ng/mL T-2 toxin. Significant differences were defined as * *p* < 0.05, *** *p* < 0.001.

Previous studies demonstrated that Nrf2 played an important role in the transcriptional activation of antioxidant genes such as heme oxygenase 1(*HO1*), NAD(P)H dehydrogenase quinone 1 (*NQO1*), glutamate-cysteine ligase modifier subunit (*GCLM*) and glutathione reductase (*GR*) [30]. To determine the role of Nrf2 in the downregulation of antioxidant genes under T-2 toxin treatment, the mRNA levels of *HO1*, *GR*, *NQO1* and *GCLM* were analyzed in Nrf2 overexpressed MCF-7 cells under T-2 toxin treatment. The results showed that T-2 toxin significantly reduced the mRNA levels of the antioxidant genes, *HO1*, *GR*, *NQO1* and *GCLM*, while overexpression of Nrf2 significantly upregulated the expression of these genes in MCF-7 cells with or without T-2 toxin treatment (Figure 3A). The importance of Nrf2 in T-2 toxin-induced downregulation of antioxidant genes was further confirmed on the protein level of HO1 by Western blot (Figure 3B,C). Furthermore, overexpression of HO1 also significantly blocked the ROS accumulation induced by 5 ng/mL T-2 toxin (Figure 3D). Therefore, these data suggested that T-2 toxin downregulated the expression of antioxidant genes by Nrf2.

### 2.4. ATF3ΔZip2a/2b Was Involved in the T-2 Toxin-Induced Ros Accumulation

**Figure 4:**
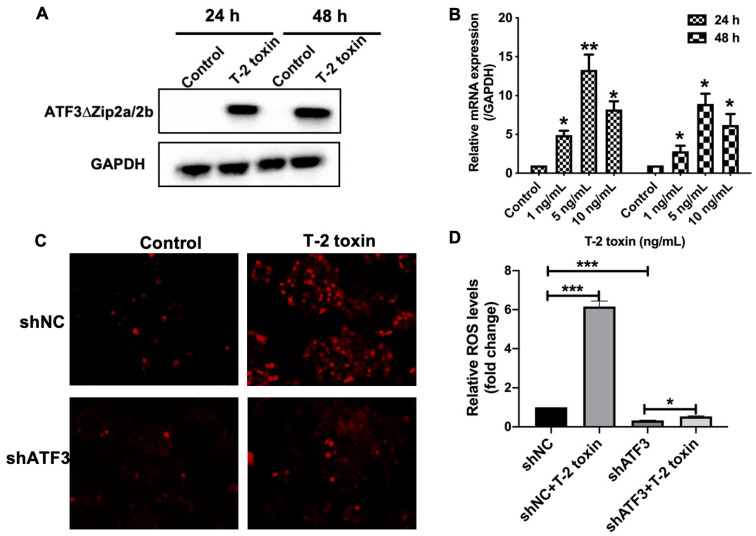
ATF3ΔZip2a/2b was involved in the ROS accumulation under T-2 toxin stress. (**A**) The Western blot analysis of ATF3ΔZip2a/2b in MCF-7 cells under 5 ng/mL T-2 toxin treatment for 24 h and 48 h. (**B**) The fold change of mRNA levels of ATF3ΔZip2a/2b under T-2 toxin treatment. (**C**,**D**) ROS levels were measured by DCFH-DA staining in ATF3 knock-down cells. shNC and shATF3 cells were treated with 5 ng/mL T-2 toxin for 24 h. Significant differences were defined as * *p* < 0.05, ** *p* < 0.01, *** *p* < 0.001.

ATF3 is an important oxidative stress-induced transcriptional factor that has been shown to activate or repress various oxidative stress-responsive genes [31]. Our previous study showed that a structurally similar mycotoxin, deoxynivalenol, significantly induced the expression of one variant of ATF3, ATF3ΔZip2a/2b [32]. Interestingly, the expression of ATF3ΔZip2a/2b was also significantly upregulated by T-2 toxin in MCF-7 cells (Figure 4A,B). To study if ATF3ΔZip2a/2b was in the ROS accumulation under T-2 toxin treatment, the ROS levels were measured in ATF3 knock–down MCF-7 cells. The DHFA-DC staining showed that ATF3 knock-down significantly attenuated the ROS accumulation in MCF-7 cells under T-2 toxin treatment (Figure 4D,E). These data indicated that ATF3ΔZip2a/2b was involved in the ROS accumulation induced by T-2 toxin.

### 2.5. T-2 Toxin Reduced the Expression of Nrf2 and Its Targeted Antioxidant Genes via Inducing ATF3ΔZip2a/2b

**Figure 5:**
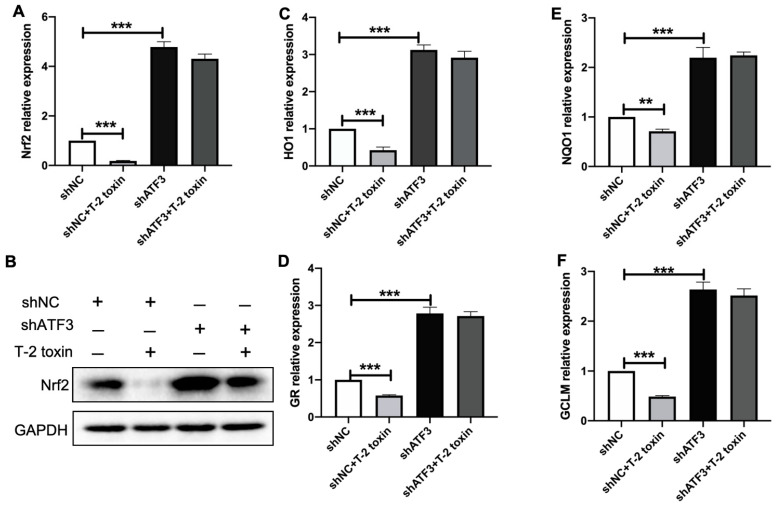
T-2 toxin downregulated the expression of Nrf2 and its downstream antioxidant genes via inducing ATF3ΔZip2a/2b. (**A**,**B**) The fold change of mRNA (**A**) and protein levels (**B**) of Nrf2 in shNC or shATF3 cells exposed to 5 ng/mL T-2 toxin for 24 h. (**C**–**F**) The fold change of mRNA levels of Nrf2 targeted antioxidant genes in shNC and shATF3 cells treated with 5 ng/mL T-2 toxin for 24 h. Significant differences were defined as ** *p* < 0.01, *** *p* < 0.001.

To study if ATF3ΔZip2a/2b was involved in the downregulation of antioxidant defense genes by T-2 toxin, the expression of Nrf2 and its targeted genes were analyzed in ATF3 knock-down MCF-7 cells. As shown in Figure 5A,B, knock down of ATF3 significantly upregulated the expression of Nrf2 by approximately four-fold in MCF-7 cells with or without treatment by 5 ng/mL T-2 toxin. Consistently, the Nrf2 targeted antioxidant genes, *HO1*, *GR*, *NQO1* and *GCLM* were also significantly upregulated by approximately four-fold in ATF3 knock-down MCF-7 cells with or without treatment by 5 ng/mL T-2 toxin (Figure 5C–F). These results suggested that T-2 toxin downregulated the expression of Nrf2 and its targeted antioxidant genes by inducing ATF3ΔZip2a/2b.

### 2.6. Overexpression of ATF3ΔZip2a/2b Promoted Ubiquitination and Degradation of Nrf2

**Figure 6:**
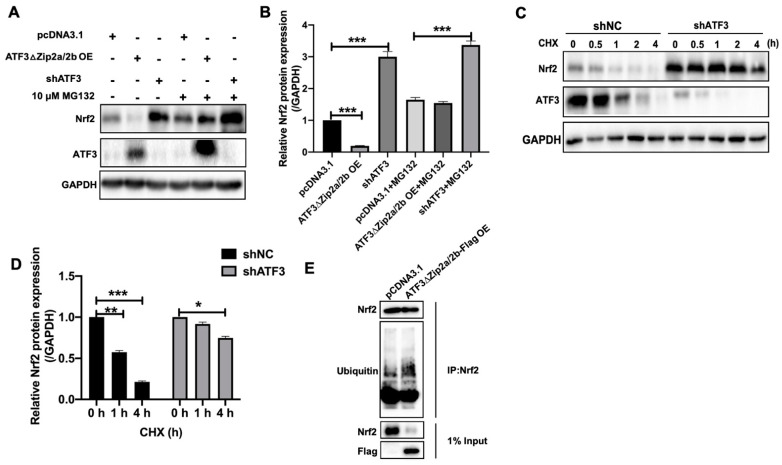
Overexpression of ATF3ΔZip2a/2b promoted the ubiquitination and degradation of Nrf2. (**A**) Western blot analysis of Nrf2 protein levels in ATF3 knock-down or ATF3ΔZip2a/2b overexpressed MCF-7 cells treated with or without MG132. (**B**) Quantitative analysis of Nrf2 protein levels in panel A as normalized to GAPDH. (**C**) Western blot analysis of the protein stability of Nrf2 in ATF3 knock-down cells. 24 h after transfection with shNC and shATF3 plasmids, MCF-7 cells were treated with protein synthesis inhibitor CHX for 0, 0.5, 1, 2 and 4 h; the protein levels of Nrf2 were detected by Western blot. (**D**) Quantitative analysis of Nrf2 protein levels in panel C. The original protein level of Nrf2 in shNC or shATF3 cells was set as 1, and the protein level of Nrf2 after CHX treatment was compared with it, respectively. (**E**) The ubiquitination of Nrf2 in ATF3ΔZip2a/2b overexpressed MCF-7 cells. The Nrf2 was immune-precipitated and analyzed using antibody against ubiquitin. Significant differences were defined as * *p* < 0.05, ** *p* < 0.01, *** *p* < 0.001.

To further clarify the mechanism of ATF3ΔZip2a/2b induced downregulation of Nrf2, the stability of Nrf2 was analyzed in ATF3ΔZip2a/2b overexpressed MCF-7 cells. As shown in Figure 6A,B, ATF3ΔZip2a/2b overexpression significantly reduced the protein level of Nrf2 by 5.5-fold, while shATF3 increased it by 3.3-fold. Interestingly, proteasome inhibitor MG132 significantly enhanced the effects of shATF3 and decreased the effects of ATF3ΔZip2a/2b overexpression on the protein level of Nrf2 (Figure 6A,B). Consistently, the degradation speed of Nrf2 in shATF3 MCF-7 cells was slower than shNC cells (Figure 6C,D). The proteins of Nrf2 were degraded to 1/2 of the original level in shNC cells, while no significant degradation in shATF3 cells 1 h after cycloheximide (CHX) treatment (Figure 6C,D). After 4 h of CHX treatment, the proteins of Nrf2 were degraded to 1/5 of the original level in shNC cells, while they were only 4/5 in shATF3 cells (Figure 6C,D). To address whether ATF3ΔZip2a/2b promoted the ubiquitination of Nrf2, the Nrf2 proteins were immune-precipitated in pcDNA3.1 or ATF3ΔZip2a/2b-Flag transfected MCF-7 cells, and their ubiquitination was analyzed using antibody against ubiquitin. The results showed that the ubiquitinated Nrf2 were increased in ATF3ΔZip2a/2b overexpressed MCF-7 cells (Figure 6E). Taken together, our data suggested that ATF3ΔZip2a/2b overexpression led to Nrf2 degradation by promoting its ubiquitination.

## 3. Discussion

As the most toxic trichothecene mycotoxin, T-2 toxin exerts a variety of toxic effects on multiple targets, including immunotoxicity, reproductive toxicity, etc. [33,34]. Previous studies showed that oxidative stress is associated with a lot of its toxic effects [35]. However, the molecular mechanism of T-2 toxin-induced oxidative stress is still unknown. In this study, we found that ATF3ΔZip2a/2b and Nrf2 acted as novel T-2 toxin targets, which are critical in T-2 toxin-induced ROS accumulation.

Previous studies have shown that oxidative stress is a critical toxicological mechanism of T-2 toxin. In mouse Leydig cells, oxidative stress played a vital role in the T-2 toxin-induced injury, which is the underpinning mechanism for T-2 toxin-induced male reproductive toxicity [36,37]. T-2 toxin treatment of N2a cells significantly decreased the activities of two key antioxidant enzymes, SOD and CAT, as well as depleting intracellular reduced GSH levels [7]. All of these studies implied that T-2 toxin could induce oxidative stress in animal cells, but there were little researches about mechanisms of T-2 toxin-induced oxidative stress, especially on human cells. Our results showed that T-2 toxin induced ROS accumulation by downregulating the expression of Nrf2 in MCF-7 cells (Figure 2). The rate-limiting enzymes in glutathione synthesis and heme catabolism that minimizes oxidative damage, such as *GCLM* and *HO1*, were downregulated after T-2 toxin treatment, and T-2 toxin also decreased the expressions of phase II detoxifying enzymes *NQO1* and *GR* (Figure 3). *GCLM*, *HO1*, *NQO1* and *GR* are well known to be the Nrf2 target antioxidant genes [18,38,39]. These data indicated that T-2 toxin increased the cellular ROS by inhibiting the Nrf2 regulated antioxidant system.

Nrf2 is considered to be an important transcription factor of cellular resistance to oxidative stress, which dominates the basal and induced expression of a wide variety of antioxidant response genes to regulate the pathophysiological and physiological progress of oxidant exposure [19,40]. Loss of Nrf2 is associated with the increased susceptibility to an array of chemical toxicities and diseases related to oxidative stress [41,42]. In osteoblastic cells, chlorogenic acid induced the Nrf2/HO1 antioxidative pathway by activating p21, which prevented dexamethasone-induced mitochondrial apoptosis [43]. It has been reported that p62 accumulation hyper-activated Nrf2 and suppressed autophagy in controlling the transcription of cellular defense enzyme genes [44]. These results indicated that the role of Nrf2 in the regulation of oxidative stress and associated physiological changes and toxicity is complex. Although Nrf2 has been reported as a pivotal regulator against T-2 toxin-induced oxidative stress [7,45], how T-2 toxin inhibited the expression of Nrf2 is still unclear. In this study, T-2 toxin significantly decreased the expression of Nrf2 in MCF-7 cells (Figure 2), which was consistent with the results in other cells [1,35,37]. Importantly, we found that the downregulation of Nrf2 under T-2 toxin stress is mediated by ATF3 (Figure 5), which provided new insight into the regulatory mechanism of Nrf2 underT-2 toxin stress.

ATF3 is associated with cellular damage and is strongly induced by multiple stress signals. Marta found that ATF3 overexpression caused depolarized mitochondria accumulation, which increased the production of mitochondrial ROS [29]. ATF3 silencing markedly suppressed the peroxisome proliferator-activated receptor-α (PPAR-α) and fat-specific protein 27 (FSP27) expression levels, which reduced fatty acid oxidation in ZDF rats [46]. The earlier research also reported that ROS induces ATF3 expression under MLN4924 treatment [47]. These studies indicated that ATF3 acted as a target for cellular oxidative stress under xenobiotics treatment and stress. Nevertheless, the mechanism of which ATF3 becomes involved in oxidative stress induced by trichothecenes has rarely been reported. Our data suggested that T-2 toxin significantly induced ATF3ΔZip2a/2b expression, and ATF3 deficiency attenuated the ROS accumulation by T-2 toxin (Figure 4), which suggested that ATF3ΔZip2a/2b is important in T-2 toxin-induced ROS production. It was reported that ATF3 acted as a repressor of the Nrf2-directed stress response pathway [26,48]. TGFβ-induced ATF3 expression, which significantly suppressed the expression of Nrf2-mediated ARE-directed phase II genes [49]. In our study, we also found that ATF3ΔZip2a/2b inhibited Nrf2 expression by promoting the ubiquitination and degradation of Nrf2 (Figure 5 and Figure 6). Previous studies have been reported that ATF3-downregulated SIRT1 and Nrf2/HO1 might play as efficient modulators of glucose metabolism and insulin synthesis via GCK downregulation in the pancrea [23,50]. These results indicated that ATF3 played an important role in Nrf2 regulation, but the mechanism remained unclear, especially for the role of ATF3 in the progress of T-2 toxin-inhibited Nrf2 expression. Our results suggested that ATF3ΔZip2a/2b-mediated ubiquitination and degradation of Nrf2 contributed to T-2 toxin-induced oxidative stress (Figure 5 and Figure 6). The Keap1-Nrf2 pathway is the known major regulator of cytoprotective responses to stresses caused by oxidative stress, but the expression of Keap1 was not changed under T-2 toxin treatment (Figure 2A), which means that ATF3ΔZip2a/2b acted as a new target in T-2 toxin-induced oxidative stress. Additionally, some researchers reported that ATF3 is a novel target of Nrf2 [50,51]. Nrf2 bound to the promoter of ATF3 to upregulate the expression of ATF3 in astrocytes which contributed to the cytoprotective function of Nrf2 against oxidative insults [51]. A possible explanation is that the regulation of Nrf2 mediated by ATF3 was a very complex process and there might exist crosstalk between ATF3 and Nrf2. ATF3 could regulate Nrf2 at protein and mRNA levels with stresses stimulation. Taken together, these results demonstrated that the impact of ATF3/Nrf2 on oxidative stress is important and complicated under different stresses.

In summary, we elaborated for the first time the molecular mechanism of T-2 toxin-induced oxidative stress and found that ATF3ΔZip2a/2b and Nrf2 acts as new targets of T-2 toxin. In particular, the role of ATF3ΔZip2a/2b in regulating Nrf2-ubiquitination had been clarified. These data provided a better understanding of the toxicology of T-2 toxin.

## 4. Materials and Methods

### 4.1. Cell Culture and Chemical Reagents

The human breast adenocarcinoma cell line MCF-7 (ATCC, HTB-22) cells were preserved in our laboratory, which were maintained in Dulbecco’s modified Eagle’s medium (DMEM, Thermo Fisher, Waltham, MA, USA) supplemented with 10% fetal bovine serum (FBS, Biological Industries, Kibbutz Beit Haemek, Israel). The MCF-7 cells were cultured at 37 °C with a 5% CO_2_ atmosphere in a constant temperature incubator. T-2 toxin, N-Acetyl-L-cysteine (NAC), Cycloheximide (CHX) and MG132 (proteasome inhibitor) were purchased from Sigma Aldrich (St. Louis, MO, USA).

### 4.2. Plasmids Construction and Cell Transfection

Based on the cDNA sequence information in GenBank of NCBI, we cloned the coding sequence (CDS) of human ATF3ΔZip2a/2b, HO1 and Nrf2 into the vector plasmid pcDNA3.1 for overexpression. The primers used in this study are listed in Table S1.

Cell transfection was conducted as previously described using Lipofectamine 3000 (Thermo Fisher, Waltham, MA, USA) according to the manufacturer’s instructions. Briefly, MCF-7 cells were seeded into 6-well plates in DMEM (10% FBS) medium and cultured to a confluence of 60–70%. The Opti-MEM (Invitrogen, Waltham, MA, USA) was used in the transfection reaction. For cell transfection, plasmid DNA was mixed with Lipofectamine 3000 by 1:1 and incubated at room temperature for 15 min. Then, the mixtures were added to the cell culture. After 5 h of culture, the transfection reagent was replaced with 500 μL fresh DMEM supplemented with 10% FBS and cultured for another 24 h. According to different experiments, different concentrations of T-2 toxin were added and cultured for 24 h or 48 h.

### 4.3. Cell Viability Assay

The cytotoxicity of T-2 toxin to MCF-7 cells was detected using Cell Counting Kit-8 (CCK-8; Yeasen, Shanghai, China). The cells were seeded in 96-well plates to a density of 1 × 10^4^ cells per well and incubated until the cells reached about 60% confluence. After being treated by T-2 toxin at indicated gradient concentrations (0–80 ng/mL) for 24 h or 48 h, 10 μL CCK-8 was added into each well and incubated for 1 h at 37 °C. 0.01% DMSO was used as a negative control. The absorbance at 450 nm was determined by a microplate reader (Spectra Max i3x, Molecular Devices, Sunnyvale, CA, USA). The half-maximal inhibitory concentration (IC_50_) was calculated according to Spearman Karber’s method [52]. All assays were repeated three times independently with six technical replicates.

### 4.4. Measurement of Intracellular Ros Levels

The intracellular ROS levels were measured using a Reactive Oxygen Species Assay Kit (Beyotime, Shanghai, China) according to the manufacturer’s instructions. Briefly, the cells were seeded into 96-well plates and exposed to various concentrations of T-2 toxin for 24 h. Following treatment, the cells were incubated with DCFH-DA for 1 h at 37 °C and measured using a microplate reader (Spectra Max i3x, Molecular Devices) with 488 nm excitation and 525 nm emission.

Dihydroethidium (DHE, Beyotime, Shanghai, China)) is used to detect the level of superoxide anion in cells. The cells were seeded into 12-well plates and exposed to various concentrations of T-2 toxin for 24 h. Following the treatment, the cells were incubated with DHE for 1 h at 37 °C and measured using a Microscope (Spectra Max i3x, Molecular Devices, Sunnyvale, CA, USA) with 488 nm excitation and 525 nm emission.

### 4.5. RNA Extraction and Quantitative RT-PCR

Total RNA was extracted using TRIzol (Life Technologies, Carlsbad, CA, USA) reagent. Quantitative RT-PCR was conducted as previously described [53]. Total RNAs (1 μg per reaction) were transcribed into complementary DNAs (cDNAs) with ReverTra Ace qPCR RT Master Mix (Toyobo, Tokoyo, Japan). Quantitative RT-PCR was perfomed using Hieff^®^ qPCR SYBR^®^ Green Master Mix (Yeasen, Shanghai, China), GAPDH was used as an internal control. The relative transcript level of each gene was computed using the 2^−^^ΔΔCt^ method [54]. Primers used in RT-PCR are listed in Table S2. All qRT-PCR assays were repeated three times independently.

### 4.6. Western Blot

The total proteins were extracted by cold RIPA lysis buffer (50 mM Tris-HCl pH 7.8, 150 mM NaCl, 1% Triton X-100) containing protease inhibitors cocktail and phosphatase inhibitors cocktail (Biomake, Houston, TX, USA). The total protein concentration of each group was detected by BCA kit [55]. Proteins were separated by SDS-PAGE and transferred onto a polyvinylidene fluoride membrane (Millipore, Boston, MA, USA). The membrane was blocked with 5% nonfat milk at room temperature for 1 h. Additionally, then the membranes were washed three times with TBST and incubated overnight at 4 °C with primary antibodies against ATF3 (1:1000, 18665S, Cell Signal Technology, Danvers, MA, USA), HO1 (1:1000, AF1333, Beyotime, Shanghai, China), Nrf2 (1:1000, AF7623, Beyotime, Shanghai, China), Ubiquitin (1:1000, P4D1-A11, Millipore, MA, USA) and GAPDH (1:1000, sc-47724, Santa Cruz, CA, USA) and then washed three times with TBST. After incubation with the secondary antibodies (1:5000, 18665S, Cell Signal Technology, MA, USA), the protein bands were developed using a Beyo ECL Star Kit (Beyotime, Shanghai, China) and visualized on a Chemi Hi Sensitivity Imaging System (BioRad, Hercules, CA, USA).

### 4.7. RNA Interference Assay

RNA interference assay was performed using lentivirus vectors including short hairpin RNA (shRNA) as described [56]. ATF3 and Nrf2 target sequences were cloned into vector pLKO.1 (Addgene, Watertown, MA, USA) and then co-transfected with packaging plasmid psPAX2 (Addgene) and envelope plasmid pMD2.G (Addgene) into MCF-7 cells. The transfected cells were treated with 5 μg/mL puromycin (Invitrogen, Waltham, MA, USA) for selection. Random sequence was used as negative control. The ATF3 and Nrf2 target sequences were GCAAAGTGCCGAAACAAGA and GGGAGGAGCTATTATCCATTC, respectively.

### 4.8. Statistical Analysis

All statistical procedures were performed using Graphpad Prism 5.0 (version 5.0, San Diego, CA, USA). Statistically significant differences among more than two groups were analyzed using the one-way analysis of variance (ANOVA). Statistical significance was defined as * *p* < 0.05, ** *p* < 0.01, *** *p* < 0.001. For every experiment, three replicates were performed and all data were expressed as the mean ± SEM. 

## Supplementary Materials

The following are available online at https://www.mdpi.com/article/10.3390/ijms22157936/s1.

## Abbreviations

ROS: reactive oxygen species; PARP-1: Poly (ADP-ribose) polymerase-1; CYBA: NADPH1 oxidase p22phox; MPO: Myeloperoxidase; Nrf2: nuclear factor erythroid 2-related factor 2; Keap1: Kelch-like ECH-associated protein 1; ATF3: activating transcription factor3; DHE: Dihydroethidium; HO1: heme oxygenase 1; NQO1: NAD(P)H dehydrogenase quinone 1; GCLM: glutamate-cysteine ligase modifier subunit; GR: glutathione reductase.
