# Supplementary material for: T-2 Toxin Induces Oxidative Stress at Low Doses via Atf3ΔZip2a/2b-Mediated Ubiquitination and Degradation of Nrf2
Source: Int J Mol Sci. 2021 Jul 25;22(15):7936. doi: 10.3390/ijms22157936 (PMC8348355; doi:10.3390/ijms22157936)
Supplement: Supplementary file 1 [file ijms-22-07936-s001.zip › ijms-1291230-supplementary.pdf]

T-2 Toxin Induces Oxidative Stress at Low Doses via Atf3ΔZip2a/2b-Mediated Ubiquitination and Degradation of Nrf2

Xiaoxuan Chen [1,2,3], Peiqiang Mu [1,2,3], Lang Zhu [1,2,3], Xiaoxiao Mao [1,2,3], Shuang Chen [1,2,3], Huali Zhong [1,2,3] and Yiqun Deng [1,2,3,*]

[1] Guangdong Provincial Key Laboratory of Protein Function and Regulation in Agricultural Organisms,
College of Life Sciences, South China Agricultural University, Tianhe District, Guangzhou 510642, China; xuanzi@scau.edu.cn (X.C.); mpeiqiang@scau.edu.cn (P.M.); 20192003022@stu.scau.edu.cn (L.Z.); mao706@stu.scau.edu.cn (X.M.); shuangchen@stu.scau.edu.cn (S.C.); 20202003035@stu.scau.edu.cn (H.Z.).

[2] Key Laboratory of Zoonosis of Ministry of Agriculture and Rural Affairs, South China Agricultural University, Guangzhou 510642, China

[3] Guangdong Laboratory for Lingnan Modern Agriculture, South China Agricultural University,
Guangzhou 510642, China

* Correspondence: yqdeng@scau.edu.cn; Tel.: +86-20-38294890; Fax: +86-20-38604987

Supplementary Table S1: Primers used for construction of expression vector in this study

Primers	Sequences
ATF3 OE-5	GGGGATCCATGATGCTTCAACACCCAGGC
ATF3 OE-3	GGGGTACCCTTGTCGTCATCGTCTTTGTAGTCTTTGCATGAGTCAACAGCCCA
HO1 OE-5	GGGGATCCATGGAGCGTCCGCAACCCGAC
HO1 OE-3	GGGGTACCCTTGTCGTCATCGTCTTTGTAGTC CATGGCATAAAGCCCTACAGC
Nrf2 OE-5	GGGGATCCATGATGGACTTGGAGCT
Nrf2 OE-3	GGGGTACCCTTGTCGTCATCGTCTTTGTAGTCCACAGTGAGTTTGCAGT

Supplementary Table S2: Primers used for quantitative real-time PCR in this study

Primers	Sequences
ATF3-RT-5	CTCCTGGGTCACTGGTGTTT
ATF3-RT-3	TTTCTCGTCGCCTCTTTTTC
CAT-RT-5	ACATGGTCTGGGACTTCTGG
CAT-RT-3	CAAGTTTTTGATGCCCTGGT
GAPDH-5	CAAGGTCATCCATGACAACTTTG
GAPDH-3	GTCCACCACCCTGTTGCTGTAG
GCLM-RT-5	TCAGTCCTTGGAGTTGCACA
GCLM-RT-3	ACACAGCAGGAGGCAAGATT
GPx-RT-5	GTCCACCGTGTATGCCTTCT
GPx-RT-3	TCTGCAGATCGTTCATCTCG
GR-RT-5	CAGTGGGACTCACGGAAGAT
GR-RT-3	AAACCCTGCAGCATTTCATC
GST-RT-5	ATGCCCATGATACTGGGGTA
GST-RT-3	GTGAGCCCCATCAATCAAGT
FOXO1-RT-5	AAGAGCGTGCCCTACTTCAA
FOXO1-RT-3	CTGTTGTTGTCCATGGATGC
HIF1A-RT-5	GAAAGCGCAAGTCCTCAAAG
HIF1A-RT-3	TGGGTAGGAGATGGAGATGC
HO1-RT-5	GTCTTCGCCCCTGTCTACTT
HO1-RT-3	CAGACAGGTCACCCAGGTAG
Keap1-RT-5	CCTTCAGCTACACCCTGGAG
Keap1-RT-5	CATGACCTTGGGGTGGATAC
NOS-RT-5	AGTTTGACCAGAGGACCCAG
NOS-RT-3	CCGTCAGTTGGTAGGTTCCT
Nrf1-RT-5	GGGCGTGAGGTTTTTGACTA
Nrf1-RT-3	GAAACTGTGCAGGGAAGCTC
Nrf2-RT-5	CCCAGCAGGACATGGATTTGA
Nrf2-RT-3	AGCTCATAGTCCTTCTGTCGC
MPO-RT-5	TGTTTGAGCAGGTCATGAGG
MPO-RT-3	CCAGATGTCGATGTTGTTGG

PARP-RT-5	GCTCCTGAACAATGCAGACA
PARP-RT-3	CATTGTGTGTGGTTGCATGA
PGC-1α-RT-5	CACCAAACCCACAGAGAACA
PGC-1α-RT-3	GGGTCATTTGGTGACTCTGG
SOD-RT-5	GGAGACTTGGGCAATGTGAC
SOD-RT-3	CACAAGCCAAACGACTTCCA
